# Application of single-step genomic evaluation using social genetic effect model for growth in pig

**DOI:** 10.5713/ajas.19.0182

**Published:** 2019-08-26

**Authors:** Joon Ki Hong, Young Sin Kim, Kyu Ho Cho, Deuk Hwan Lee, Ye Jin Min, Eun Seok Cho

**Affiliations:** 1 National Institute of Animal Science, Rural Development Administration, Cheonan 31000, Korea; 2 Department of Animal Life Resources, Hankyong University, Anseong 17579, Korea

**Keywords:** Accuracy, Breeding Value, Landrace, Single-step Genomic Best Linear Unbiased Prediction (BLUP), Social Genetic Effects, Yorkshire

## Abstract

**Objective:**

Social genetic effects (SGE) are an important genetic component for growth, group productivity, and welfare in pigs. The present study was conducted to evaluate i) the feasibility of the single-step genomic best linear unbiased prediction (ssGBLUP) approach with the inclusion of SGE in the model in pigs, and ii) the changes in the contribution of heritable SGE to the phenotypic variance with different scaling ω constants for genomic relationships.

**Methods:**

The dataset included performance tested growth rate records (average daily gain) from 13,166 and 21,762 pigs Landrace (LR) and Yorkshire (YS), respectively. A total of 1,041 (LR) and 964 (YS) pigs were genotyped using the Illumina PorcineSNP60 v2 BeadChip panel. With the BLUPF90 software package, genetic parameters were estimated using a modified animal model for competitive traits. Giving a fixed weight to pedigree relationships (τ: 1), several weights (ω_xx_, 0.1 to 1.0; with a 0.1 interval) were scaled with the genomic relationship for best model fit with Akaike information criterion (AIC).

**Results:**

The genetic variances and total heritability estimates (*T*^2^) were mostly higher with ssGBLUP than in the pedigree-based analysis. The model AIC value increased with any level of ω other than 0.6 and 0.5 in LR and YS, respectively, indicating the worse fit of those models. The theoretical accuracies of direct and social breeding value were increased by decreasing ω in both breeds, indicating the better accuracy of ω_0.1_ models. Therefore, the optimal values of ω to minimize AIC and to increase theoretical accuracy were 0.6 in LR and 0.5 in YS.

**Conclusion:**

In conclusion, single-step ssGBLUP model fitting SGE showed significant improvement in accuracy compared with the pedigree-based analysis method; therefore, it could be implemented in a pig population for genomic selection based on SGE, especially in South Korean populations, with appropriate further adjustment of tuning parameters for relationship matrices.

## INTRODUCTION

The genetic effect of an individual on the phenotypes of its social partners (i.e., pen mates) is often termed the social genetic effect (SGE) or the indirect genetic effect [1]. The growth rate is a key trait in pig breeding goals because it contributes to economic efficiency. However, negative effects of social interactions, such as tail biting, or excessive aggression can inhibit growth of pen mates, resulting in reduce productivity in pig farming. The report by Bergsma et al [2] on pigs indicated that the heritable social interaction among various group members might play a role in their average daily gain (ADG). In this regard, Bijma et al [1] stated that the total breeding value (TBV), expressed as the combined direct breeding value (DBV) of an individual and social breeding values (SBV) of pen mates, for growth performance can be used in selection that includes SGE. Although the estimation of SBV in pigs is achievable through the traditional pedigree-based approach directly using phenotypes, this will inevitably result in lower accuracy [3]. Therefore, it is also highly desirable to use a model i.e., genomic best linear unbiased prediction (BLUP) [4], for the selection of pigs which will include SGE as well as improve accuracy in predictions of breeding values.

In recent years, a single-step genomic BLUP (ssGBLUP) which provides better predictions for all animals in a pedigree [5], is a method of choice for genomic evaluation. In pigs, Christensen et al [6] has provided evidence of improved predictions through ssGBLUP, compared with the pedigree-based method. They also suggested an adjustment of the genomic relationship matrix with ssGBLUP. As genomic information is now often used to optimize selection outcomes, it seems reasonable that the inclusion of social interactions alongside genomic information might benefit the selection of in Korean pigs as well. Therefore, this study was established with the following aims: i) to verify the feasibility of the SGE model using ssGBLUP in dam lines and ii) to evaluate the influences of different scales of matrices on the contribution of heritable social effects to the phenotypic variance.

## MATERIALS AND METHODS

### Animal phenotypes

The phenotypic dataset on the growth rate of animals was obtained through performance tests of Yorkshire (YS; n = 21,762) and Landrace (LR; n = 13,166) pigs between 2005 and 2015. These pigs were born and raised in a closed nucleus (breeding) farm in South Korea. Animals were reared in pens where each pen housed 4 to 10 YS or 3 to 8 LR pigs of the same sex. Each group of animals comprised 1 to 7 YS or 1 to 6 LR full-sibs. The performance evaluations on the ADG of pigs started soon after each animal reached a live body weight of 30 kg, and were finished when a target weight of 90 kg was attained. On average, fewer than 160 days were required to attain this target weight. The animal phenotypes selected to be studied were based on the size of the group (frequency >10%). The average ADG was recorded to be 787.9±88.3 g/d in LR and 792.5±92.1 g/d in YS.

Complete pedigrees of the studied animals were obtained from the Korean Animal Improvement Association database. The total numbers of animals in the pedigree of the two breeds were 14,013 (LR) and 22,536 (YS). The numbers of individuals for which both parents were known were 13,916 (LR) and 22,324 (YS). In the whole pedigree, about 96% (LR) and 62% (YS) of the animals were inbred. The average inbreeding coefficients were 0.035 and 0.015 in LR and YS, respectively. The ranges of inbreeding coefficients were 0.0001 to 0.263 (YS) and 0.001 to 0.274 (LR). The observed average family sizes were 3.97 and 4.11 in LR and YS, with ranges of 2 to 15 and 2 to 17, respectively. The population structures of these breeds were determined using the CFC v1.0 software package [7].

The experimental protocols describing the management and care of the animals were reviewed and approved according to the guide for the care and use of laboratory animals (National Institute of Animal Science, Animal Care Committee of Korea) on 7 March 2014 (approval number: NIAS 2014-289). The management practices on the studied population stated that each pen was 2.5×3.6 m (~0.9 m^2^/pig) in size with solid concrete flooring. Pigs were fed *ad libitum* and water was constantly accessible through nipple drinkers. The feeding program was applied in accordance with pig testing standards of the Korean Animal Improvement Association (http://www.aiak.or.kr/eng/index.jsp).

### Genotyping of animals

The genomic DNA of pigs was extracted from their blood samples using a standard protocol. A total of 2,005 pigs from both LR (1,041) and YS (964) breeds were genotyped using the Illumina PorcineSNP60 v2 BeadChip panel, which comprised 61,565 SNP (single nucleotide polymorphism) markers [8]. The quality control (QC) procedure for the genotype data included the deletion of individuals with pedigree errors, removal of monomorphic SNP genotypes, SNPs on sex chromosomes or SNPs with minimum allele frequencies (<0.9), genotype call rate of <0.90, animal missing rate of >0.90, Hardy-Weinberg equilibrium of 0.15, and the SNPs with displaced segregation distortion [9,10]. After QC, the final dataset contained genotypes from a total of 1,915 pigs (LR, 1,029; YS, 886). The total number of autosomal SNPs was reduced to 37,779 in LR and 41,503 in YS, respectively. The correlations of off-diagonal elements of **G** and **A**_22_ matrices were 0.75 and 0.76 for LR and YS, respectively.

### Statistical analysis

#### Estimation using pedigree information

The variances and covariances of the studied traits were estimated by an animal model applying the REML approach. The effects of batch, sex, and group size were fitted as fixed effects. In the model, age at target weight was fitted as a covariate effect. The effect of batch was formed by combining year, month, and week based on each performance test. The models also included two nongenetic random effects, namely, birth litter and group effects [1,11]. To take into account differences in group size and to prevent the overestimation of heritable variances for SGE, an additional covariate term known as dilution, ${\left(\frac{\text{Average group size}-1}{\text{Group size}-1}\right)}^{0.5}$ [12], was added to the SGE [3,11,13]. Animals were fitted as a random effect in the model. The BLUPF90 software package Misztal et al [14] was used for the estimation of parameters by fitting a classical model with pedigree relationships only (PED_classic_) and a social model pedigree relationships only modified for competitive traits (PED_social_) [11] as follows:

$$\begin{array}{l} y=Xb+Z_{\text{D}a\text{D}}+Wl+Vg+e\;({\text{PED}}_{\text{classic}})\\ y=Xb+Z_{\text{D}a\text{D}}+Z_{\text{S}a\text{S}}+Wl+Vg+e\;({\text{PED}}_{\text{social}}) \end{array}$$

where **y** is the vector of observations (ADG), *b* is the vector of fixed effects, *a*_D_ is the vector of random direct additive genetic effects, *a*_S_ is the vector of random SGEs, *l* is the vector for random birth litter, *g* is the vector of random group, and *e* is the vector of residuals. **X**, **Z**_D_, **Z**_S_, **W**, and **V** are the corresponding incidence matrices. Assumptions for the probability distributions were $g\sim \text{N}\left(0,I\sigma_{g}^{2}\right),\;l\sim \text{N}\left(0,I\sigma_{l}^{2}\right),\;c\sim \text{N}\left(0,I\sigma_{c}^{2}\right)$, and $e\sim \text{N}\left(0,I\sigma_{e}^{2}\right)$, in which N( ) indicates a normal distribution; **I** is an identity matrix of appropriate dimensions; and $\sigma_{g}^{2},\sigma_{l}^{2},\sigma_{c}^{2}$, and $\sigma_{e}^{2}$ are the variances of the corresponding effects. In Model 1, direct additive genetic effects had the following distribution: $a_{D}\sim N(0,A\sigma_{a_{D}}^{2})$, in which **A** is the numerator relationship matrix and $\sigma_{{\text{a}}_{\text{D}}}^{2}$ is the variance of direct additive genetic effects. In Model 2, direct and indirect additive genetic effects had the following multivariate normal (MVN) distribution: $\left[\begin{array}{c} a_{D}\\ a_{S} \end{array}\right]\sim \text{MVN }(0,C⊗A)$, in which **C** is defined by the matrix $\left[\begin{array}{cc} \sigma_{a_{D}}^{2} & \sigma_{a_{D}a_{S}}\\ \sigma_{a_{D}\;a_{S}} & \sigma_{a_{S}}^{2} \end{array}\right],\sigma_{a_{S}}^{2}$ is the variance of indirect genetic effects, *σ_a_D_a_S__* is the covariance between direct and indirect genetic effects, and **C**⊗**A** denotes the Kronecker product of two matrices.

According to Bijma et al [1] for traits affected by heritable social effects, the variance of TBV represents the total heritable variation that is exploitable for selection. The TBV of the *i*th animal is defined as follows:

$${TBV}_{i}=a_{D,i}+(n-1)a_{S,i}$$

The TBV is the heritable effect of an individual on trait values in the population, which is the sum of its direct genetic effect (*a_D_*_,_*_i_*) on its own phenotype and its (*a_S_*_,_*_i_*) on the phenotypes of its *n* – 1 group mates. Bijma et al [1] also stated that the total heritable variance determines the population’s potential in response to selection and can be expressed as:

$$\sigma_{TBV}^{2}=\sigma_{a_{D}}^{2}+2(n-1)\sigma_{a_{D}\;a_{S}}+{\left(n-1\right)}^{2}\sigma_{a_{S}}^{2}$$

According to Bergsma et al [2], the phenotypic variance for such a model can be calculated as follows:

$$\sigma_{P}^{2}=\sigma_{a_{D}}^{2}+(n-1)\sigma_{a_{S}}^{2}+\sigma_{g}^{2}+\sigma_{l}^{2}+\sigma_{e}^{2}$$

where n indicates the average size of social groups. The total heritable variance can be expressed relative to phenotypic variance [2] as follows:

$$T^{2}=\sigma_{TBV}^{2}/\sigma_{P}^{2}$$

#### Estimation using single-step method

The relationship matrix **H**, in single-step evaluation, defines the relationship among genotyped and nongenotyped animals. The inverse of the **H** matrix is rather simple in structure [15,16] and can be given as:

$$H^{-1}=A^{-1}+\left[\begin{array}{cc} 0 & 0\\ 0 & G^{-1}-A_{22}^{-1} \end{array}\right]$$

where **A**_22_ is the matrix for genotyped animals only (a submatrix derived from the pedigree-based relationship matrix, **A** and **G** is the relationship matrix among individuals based on genomic information. The **G** matrix was constructed according to VanRaden [4]. Both **A**_22_ and **G** matrices were subsequently combined. Thus, overall, the two matrices represented similar diagonals. However, the mixed model equations for a single step mainly differed from the pedigree-based model by a matrix block, τ (0.95 G+0.05 A_22_)^−1^ – ω A_22_^−1^, given to the genotyped animals [17,18]. The constant ω represents the proportion of polygenic variances that were unexplained by markers. The parameters τ and ω scaled the size of the genomic and pedigree relationships, respectively. The weights for the ω parameter were between 0.1 and 1.0, whereas τ was fixed at 1. The models including genomic information are denoted as ω_1.0_, ω_0.9_, ω_0.8_, ω_0.7_, ω_0.6_, ω_0.5_, ω_0.4_, ω_0.3_, ω_0.2_, and ω_0.1_, in accordance with their values of the constant ω, whereas a model with pedigree information only is denoted as PED in later sections.

#### Validation process

Accuracy of breeding value was calculated in two different ways (theoretical accuracy [3] and cross validation [6]). The last 2 years were masked as the validation data set and predictions were made using the first 9 years as the training data set. The validation data set for LR and YS contained 10% and 8% of the observations, respectively. The theoretical accuracy of the estimated breeding value for the *i*th individual with the *m*th model was calculated as follows:

$$R_{i,m}=\sqrt{1-\frac{{PEV}_{i,m}}{(1+F_{i})\;\sigma_{m}^{2}}}$$

where *PEV* is the prediction error variance of its breeding value, *F* is the inbreeding coefficient of an individual as computed from the pedigree, and *σ*^2^ is the additive genetic variance of the model. We also calculated correlation between corrected phenotype and the combined breeding value (CBV) for the validation pigs. Accuracy was defined as

$$r=(\text{cor}[\text{CBV},y_{\text{c}}])$$

where CBV is the sum of pig’s own direct breeding value and SBVs of pen mates, *y_c_* is corrected ADG for fixed effects.

## RESULTS AND DISCUSSION

### Model fitness

The variances, covariances, and various model parameters obtained from the studied models for LR and YSs are presented in Tables 1 and 2, respectively. The Akaike information criterion (AIC) parameter of the pedigree-classical model was higher than the pedigree-social model in both breeds. This result showed that model including SGE fitted the data significantly better than a classical animal model. In addition, AIC parameter of the pedigree-social model was the highest in both breeds compared with those of all ssGBLUP methods. The AIC as an indicator of the goodness fit of the models indicates that the ssGBLUP models performed better in general, which was as expected due to the addition of genomic information alongside the pedigree relationship. This is a feasible approach with a single-step method as it provides more accurate predictions for both genotyped and nongenotyped animals [6,11,18,19]. Therefore, a ssGBLUP analysis including SGE in the model would be a better choice for the prediction of traits in pigs. However, differences were observed among the various model fits with different scaling factors in the single-step methods. Among the ssGBLUP models, the model with ω of 1.0 showed the worst fit, regardless of the breed. The best fitting models in this study were those with ω_0.6_ and ω_0.5_ in LR (Table 1) and YR (Table 2), respectively, as indicated by them having the lowest AIC estimates. The model AIC value increased with any level of ω other than 0.6 and 0.5 in LR and YS, respectively, indicating the worse fit of those models. Our results obtained through testing different levels of ω (0.1 to 1.0) indicate that a ssGBLUP method essentially relies on tuning the scales of matrices related to pedigree and genotype relationships, which will lead to less biased model estimates [6,15,17,20–22]. This study strongly coincides with many previous reports in that the choices of appropriate levels of constants (τ and ω) are rather arbitrary, and are to be determined through fine tuning. For instance, Misztal et al [17] reported the best combination of τ = 1.5 and ω = 0.6 in their study on dairy cattle. Another study in dairy cattle by Harris et al [23] also used both parameters at levels as low as 0.5. Likewise, Koivula et al [20] reported using various combinations of **A** and **G** matrices to find the best option in their study. In pig, Christensen et al [6] suggested a single-step method that is adjusted for the genomic relationship matrix. In another study by Misztal et al [24], a model with slower convergence at ω values greater than 1 was reported, as their **H** matrix was found to be nonpositive at higher values of this constant. In this context, it is crucial to find appropriate scaling parameters that will ensure better accuracy, lower bias, and easier convergence. It is also important to consider appropriate weights for relationship matrices through scaling factors as any smaller constant for ω is likely to decrease the emphasis on the genomic relationships and increase the importance of the pedigree relationships [20]. This might explain our estimates obtained with levels of ω lower than those in best fit models, where model estimates might have been associated with some biases due to the lower weight in genotyped animals through their genomic relationships.

### Genetic parameters

The genetic variances and total heritability estimates (*T*^2^) were mostly higher with ssGBLUP than in the pedigree-based analysis (Tables 1, 2). Among the ssGBLUP models, the genetic variances and *T*^2^ were increased by decreasing ω in both breeds. Therefore, the *T*^2^ of ω_0.1_ model was the highest in both breeds (LR, 0.64; YS, 0.88). The best single-step models (ΔAIC = 0) showed larger estimates of direct and social variances than pedigree-based methods, and thus also larger covariance estimates, resulting in higher total heritability estimates with those models. The *T*^2^ estimates with the best fitting models were 0.54 and 0.80 in LR and YS, respectively. They were also greater than those of the pedigree-based analysis method by 0.13 and 0.22 in these two breeds, respectively. However, our *T*^2^ estimates for LR with the ω_10_ model coincided strongly with those of Bergsma et al [25] and Duijvesteijn [3]. Comparing the breeds, both direct and social genetic contributions were higher in YS than in LR, so their *T*^2^ estimates also exhibited the same trend. Note that even when the social variance is markedly smaller than direct genetic variance, its contribution to $\sigma_{\text{TBV}}^{2}$ would be substantial due to the factor (n–1)^2^, especially when group sizes are large, as was the case with YS. The lower *T*^2^ estimates in LR could also be due to the larger nongenetic litter effects and negative covariances between direct and social effects. According to Bijma et al [1], the positive covariance between direct and social genetic variances is likely to increase the total heritable variation, which coincides well with the present study. The correlation coefficients between DBV and SBV were somewhat weaker in LR (−0.05 to 0.09) than in YS (0.28 to 31). Some earlier reports [2,3,26] also stated somewhat similar correlations, mostly positive but not significant. In this study, the positive correlation in YS could indicate that their pen mates might also have stimulated a greater ADG. Given that SBV is passed on to pen mates, the positive genetic correlation between the direct and associative effects indicates that pigs with a high DBV will also have a high SBV. In other words, the YR pigs in our study may show more positive responses to selection for social interactions than the LR pigs. Nonetheless, breed differences for social interactions are not unlikely. Bergsma et al [2] suggested that the absence of conflict between an individual’s own growth and mate growth might be a consequence of neutral or slightly cooperative social interactions. For the negative or neutral associative effects in LR pigs in this study, it is possible that these pigs were in less competition for food and growth as the amount of space that each of them had on average (3 to 8 pigs/9 m^2^ pen) was lower than that of YR (4 to 10 pigs/9 m^2^ pen).

### Validation

Table 3 illustrates the accuracy for breeding values obtained with different models. The levels of theoretical accuracy obtained for DBV with PED_classic_ and PED_social_ models in each breed were same and also the lowest among the different models (LR, 0.52; YS, 0.55). The ω_1.0_ models also performed poorly in DBV prediction (LR, 0.55; YS, 0.58). Among the ssGBLUP models, the theoretical accuracy of DBV was increased by decreasing ⌐ω in both breeds (LR, 0.55 to 0.66; YS, 0.58 to 0.64). The best fit models based on AIC exhibited an increase of accuracy by 5% to 8% compared with the ω_1.0_ models in both breeds. The ranges of SBV accuracies with the PED_social_ in LR and in YS were 0.16 and 0.31, respectively. Similar to DBV, both PED_social_ and ω_1.0_ models performed poorly in SBV prediction. However, unlike the DBVs from the single-step methods, the best fitting models exhibited notable increases in SBV accuracies by 39% (LR) and 19% (YS) with ω_0.6_ and ω_0.5_, respectively, compared with each of the breed’s worst fit (ω_1.0_) model. In cross validation, the correlations between CBV and corrected phenotype were also mostly higher with ssGBLUP than in the pedigree-based analysis. However, there were little differences among the ssGBLUP models. The ranges of correlations between CBV and corrected phenotype in LR and YS were 0.31 to 0.33 and 0.21 to 0.22, respectively. The correlative prediction methods showed more variability in terms of ranking of models across traits and replicates so care should be taken interpreting these results with small sample sizes [27]. Putz et al [27] also suggested that for within-breed selection, theoretical accuracy using the prediction error variance was consistent and accurate in ssGBLUP. However, selection programmes should be careful which validation method they choose and should inspect multiple methods if possible [27]. Therefore, to minimize AIC and to increase theoretical accuracy in this study, the optimal values of ω in LR and YS were 0.6 and 0.5, respectively. Martini [28] reported that increasing τ or decreasing ω may mainly decrease inflation by decreasing the variance of the estimated breeding values, which indicate the possibility of further adjustment of τ in the **H** matrix.

### Prospect of social genetic effects

The phenotypic variability of some traits that are expressed in the social environment could be significantly influenced by SGEs. Earlier reports on such traits, for instance, social dominance or aggressiveness, also suggested that SGEs can substantially influence total phenotypic variability [29–32]. The importance of SGEs can also be recognized from many previous reports [33–35], which showed that the higher SBV and some desirable characteristics in pigs i.e., fearlessness, stress-tolerance are associated to each other. These characteristics in commercial pig production are particularly beneficial for ease of farm management. For this reason, appropriate attention to such socially influenced traits alongside the pig population structure is vital when genomic selection is considered [36]. Certain strategies could also be applied during selection to achieve a high SBV for a desirable trait. One such approach is to select animals with higher TBVs to improve group performance, especially for growth traits [1–3]. Direct selection of pigs for SBV could be another strategy to alter their social behavior. Earlier evidence suggested that high SBV, due to apathy of the animal, could reduce negative social effects on the growth of others [37–39]. Moreover, the inclusion of SNP effects with SGEs in the model could provide better predictions [40]. For successful realization of TBV, it is also important to consider social environments, such as the mixing method of suckling piglets [41].

## CONCLUSION

For SGEs, our study showed greater improvement in parameter estimates through ssGBLUP over the traditional pedigree-based method. Both breeds differed to some extent for their estimated parameters. The value of ω used for adjusting **A**_22_ matrix also differed between the best fitting models for the LR and YS breeds. But it was clear that the models with ω of 1.0 in the **H** matrix were the worst fitting. Our study also indicated the possibility of further adjustment of other model parameters (α, β, τ) in the **H** matrix to reduce inflation of the estimated breeding values. Our results also indicated the value of further analysis with a greater sample size to obtain a more robust estimation of breeding values. We believe that our results provide useful insights for future modeling of SGE in the genomic selection of pig breeds, especially in South Korea.

## Figures and Tables

**Table 1:** Estimates of variances, covariances, genetic parameters, and accuracies for different models in Landrace pigs

Method	$\sigma_{a_{D}}^{2}$	*σ*_*a*_*D*_*a*_*S*__	$\sigma_{a_{S}}^{2}$	$\sigma_{g}^{2}$	$\sigma_{l}^{2}$	$\sigma_{e}^{2}$	*r*	$\sigma_{p}^{2}$	*T*^2^	ΔAIC
PED_classic_	2,078	-	-	433	208	3,039	-	5,758	0.36	72.9
PED_social_	2,069	3	14	383	208	3,037	0.02	5,825	0.41	71.5
ω_1.0_	2,136	−6	9	410	210	2,999	−0.05	5,840	0.39	19.8
ω_0.9_	2,392	−2	14	395	204	2,883	−0.01	5,997	0.44	6.9
ω_0.8_	2,589	2	17	382	201	2,796	0.01	6,122	0.48	1.8
ω_0.7_	2,747	7	20	372	200	2,728	0.03	6,226	0.52	0.1
ω_0.6_	2,878	11	23	362	200	2,673	0.04	6,316	0.54	0.0
ω_0.5_	2,988	15	25	353	200	2,628	0.05	6,395	0.57	1.0
ω_0.4_	3,081	19	27	344	201	2,590	0.06	6,463	0.59	2.8
ω_0.3_	3,162	22	30	336	202	2,558	0.07	6,524	0.61	4.9
ω_0.2_	3,233	26	31	329	203	2,532	0.08	6,580	0.62	7.5
ω_0.1_	3,294	29	33	322	204	2,509	0.09	6,630	0.64	10.3

$\sigma_{a_{D}}^{2}$, direct genetic variance; *σ*_*a*_*D*_*a*_*S*__, covariance between direct and social genetic effects; $\sigma_{a_{S}}^{2}$, social genetic variance; $\sigma_{g}^{2}$, random group variance; $\sigma_{l}^{2}$, random litter variance; $\sigma_{e}^{2}$, random residual variance; *r*, correlation between direct and social genetic effects; $\sigma_{p}^{2}$, phenotypic variance; *T*^2^, total heritability for model including social genetic effects; ΔAIC, change in Akaike’s information criteria from the best (minimum) model; PED_classic_, the classic model with pedigree relationships only; PED_social_, the social model with pedigree relationships only; ω_xx_, the model with weighted $A_{22}^{-1}$ matrix by different ω constants.

**Table 2:** Estimates of variances, covariances, genetic parameters, and accuracies for different models in Yorkshire pigs

Method	$\sigma_{a_{D}}^{2}$	*σ*_*a*_*D*_*a*_*S*__	$\sigma_{a_{S}}^{2}$	$\sigma_{g}^{2}$	$\sigma_{l}^{2}$	$\sigma_{e}^{2}$	*r*	$\sigma_{p}^{2}$	*T*^2^	ΔAIC
PED_classic_	2,255	-	-	675	260	3,712	-	6,901	0.33	102.1
PED_social_	2,320	72	23	479	256	3,739	0.31	7,084	0.58	81.0
ω_1.0_	2,323	69	23	483	268	3,732	0.30	7,092	0.57	24.7
ω_0.9_	2,565	79	29	453	262	3,621	0.29	7,260	0.64	12.2
ω_0.8_	2,763	86	33	430	258	3,530	0.28	7,395	0.69	5.8
ω_0.7_	2,930	93	37	412	256	3,454	0.28	7,511	0.73	2.4
ω_0.6_	3,074	99	40	396	255	3,389	0.28	7,611	0.77	0.6
ω_0.5_	3,201	104	43	383	254	3,332	0.28	7,700	0.80	0.0
ω_0.4_	3,313	109	45	372	253	3,282	0.28	7,778	0.82	0.2
ω_0.3_	3,414	113	47	361	253	3,238	0.28	7,851	0.84	0.9
ω_0.2_	3,504	117	49	352	254	3,198	0.28	7,915	0.86	2.0
ω_0.1_	3,585	121	51	343	254	3,163	0.28	7,975	0.88	3.5

$\sigma_{a_{D}}^{2}$, direct genetic variance; *σ*_*a*_*D*_*a*_*S*__, covariance between direct and social genetic effects; $\sigma_{a_{S}}^{2}$, social genetic variance; $\sigma_{g}^{2}$, random group variance; $\sigma_{l}^{2}$, random litter variance; $\sigma_{e}^{2}$, random residual variance; *r*, correlation between direct and social genetic effects; $\sigma_{p}^{2}$, phenotypic variance; *T*^2^, total heritability for model including social genetic effects; ΔAIC, change in Akaike’s information criteria from the best (minimum) model; PED_classic_, the classic model with pedigree relationships only; PED_social_, the social model with pedigree relationships only; ω_xx_, the model with weighted $A_{22}^{-1}$ matrix by different ω constants.

**Table 3:** The accuracy of estimated breeding values for different models in pigs

Method	Landrace			Yorkshire		
	
	DBV_acc_	SBV_acc_	*Cor*	DBV_acc_	SBV_acc_	*Cor*
PED_classic_	0.52 (0.06)	-	0.27	0.55 (0.04)	-	0.20
PED_social_	0.52 (0.06)	0.16 (0.05)	0.28	0.55 (0.04)	0.31 (0.03)	0.21
ω_1.0_	0.55 (0.09)	0.11 (0.09)	0.33	0.58 (0.05)	0.33 (0.04)	0.21
ω_0.9_	0.58 (0.07)	0.33 (0.09)	0.33	0.59 (0.05)	0.42 (0.04)	0.21
ω_0.8_	0.60 (0.07)	0.42 (0.07)	0.33	0.60 (0.05)	0.46 (0.05)	0.22
ω_0.7_	0.61 (0.07)	0.47 (0.07)	0.32	0.61 (0.05)	0.49 (0.05)	0.22
ω_0.6_	0.62 (0.07)	0.50 (0.07)	0.32	0.62 (0.06)	0.51 (0.06)	0.22
ω_0.5_	0.63 (0.07)	0.52 (0.07)	0.32	0.63 (0.06)	0.53 (0.06)	0.22
ω_0.4_	0.64 (0.07)	0.54 (0.07)	0.32	0.63 (0.06)	0.54 (0.06)	0.22
ω_0.3_	0.65 (0.07)	0.56 (0.07)	0.32	0.64 (0.06)	0.55 (0.06)	0.22
ω_0.2_	0.65 (0.07)	0.57 (0.08)	0.31	0.64 (0.06)	0.56 (0.06)	0.22
ω_0.1_	0.66 (0.07)	0.58 (0.08)	0.31	0.64 (0.06)	0.57 (0.07)	0.22

DBV_acc_, the theoretical accuracy of direct breeding value; SBV_acc_, the theoretical accuracy of social breeding value; Cor, the correlation between corrected phenotype and the combined breeding value (CBV); PED_classic_, the classic model with pedigree relationships only; PED_social_, the social model with pedigree relationships only; ω_xx_, the model with weighted $A_{22}^{-1}$ matrix by different ω constants.
